# SeqAfrica: empowering Africa’s fight against antimicrobial resistance through genomics

**DOI:** 10.3389/fpubh.2025.1716498

**Published:** 2025-12-17

**Authors:** Pernille Nilsson, Christa Twyford Gibson, Natasia R. Thornval, Niamh Lacy-Roberts, Christina Odgaard, Christian Owusu-Nyantakyi, Grebstad Rabbi Amuasi, William Boateng, Quaneeta Mohktar, Alfred Bortey, Erkison Ewomazino Odih, Gabriel Temitope Sunmonu, Happiness H. Kumburu, Tolbert Sonda, Jinal N. Bhiman, Daniel G. Amoako, Mignon du Plessis, Bright Adu, Marco van Zwetselaar, Anne von Gottberg, Blandina T. Mmbaga, Iruka N. Okeke, Anthony M. Smith, Beverly Egyir, Rene S. Hendriksen

**Affiliations:** 1 Technical University of Denmark, National Food Institute, Research Group for Global Capacity Building, Kgs. Lyngby, Denmark; 2 Noguchi Memorial Institute for Medical Research, College of Health Sciences, University of Ghana, Accra, Ghana; 3 Department of Pharmaceutical Microbiology, Faculty of Pharmacy, University of Ibadan, Ibadan, Nigeria; 4 Kilimanjaro Clinical Research Institute, Moshi, Tanzania; 5 Centre for Respiratory Diseases and Meningitis, National Institute for Communicable Diseases, a division of the National Health Laboratory Service, Johannesburg, South Africa; 6 Department of Clinical Microbiology and Infectious Diseases (CMID), School of Pathology, Faculty of Health Sciences, University of the Witwatersrand, Johannesburg, South Africa; 7 School of Medicine, KCMC University, Moshi, Tanzania; 8 Division of the National Health Laboratories Service, Centre for Enteric Diseases, National Institute for Communicable Diseases, Johannesburg, South Africa; 9 Department of Medical Microbiology, Faculty of Health Sciences, University of Pretoria, Pretoria, South Africa

**Keywords:** Africa, antimicrobial resistance, low- and middle-income countries, whole genome sequencing, surveillance, capacity building

## Abstract

The ongoing threat of antimicrobial resistance (AMR) demands capacity strengthening in Africa for improved pathogen surveillance. The high-resolution picture of AMR provided by pathogen whole genome sequencing (WGS) can help close data gaps and inform disease prevention strategies, interventions and public health actions. Here, we report on phase 1 of the Fleming Fund-supported SeqAfrica project (2019–2023), one of the first genomic AMR surveillance networks in Africa. SeqAfrica established five regional sequencing hubs across West, East, and Southern Africa, expanded infrastructure, and delivered hybrid training programs to strengthen workforce capacity. During phase 1, the network generated 29,269 pathogen genomes (18,264 bacterial, 300 fungal, and 10,705 SARS-CoV-2) from 21 African countries, contributing to 40 scientific publications and substantial genomic data for national and global surveillance efforts, supporting outbreak investigations and antimicrobial stewardship initiatives. The median turnaround time from sample receipt to data release was 12 weeks (range: 3–104 weeks), demonstrating the feasibility of genomic AMR surveillance despite logistical challenges. By nurturing a community of practice, expanding the workforce, and translating data into actionable insights, SeqAfrica has advanced the integration of pathogen genomics into national and regional surveillance frameworks. However, sustaining this capacity remains a challenge amid global funding constraints, procurement bottlenecks, and workforce retention issues. Lessons learned from implementation include successes in regional collaboration and persistent challenges in procurement, workforce retention, and metadata completeness, which informed the design of phase 2. As Africa continues to invest in genomic health infrastructure, SeqAfrica provides a proven model for embedding pathogen genomics into public health strategies and strengthening AMR surveillance across the continent.

## 1 Introduction

Antimicrobial resistance (AMR) is a global health issue affecting human, animal, and environmental health as well as food safety with low- and middle-income countries (LMICs), which bear the heaviest consequences of drug-resistant infections (1). An estimated 1.27 million deaths per year were directly attributable to AMR in 2019 making it the leading contributor of global deaths (1). Improving surveillance of AMR microorganisms is one of the four major strategic objectives in the Africa Union Continental Framework for AMR Control 2020–2025 (2). The African Union Regional Quadripartite Collaboration (FAO, UNEP, WHO and WOAH) aims to strengthen, harmonize, and enhance AMR surveillance and laboratory capacities through a One Health approach. Since the endorsement of the WHO AMR Global Action Plan, many countries have developed National Action Plans (NAPs) on AMR, including interventions to optimize surveillance of infectious diseases and drug-resistant infections (3, 4). In 2019, only 13 countries in the African region had developed NAPs, and while this had increased to 39 countries by the end of 2024, implementation of AMR interventions in Africa has been limited (5, 6). AMR surveillance systems are often fragmented and uncoordinated (7, 8), and few countries have implemented comprehensive surveillance systems, resulting in significant gaps in the AMR data landscape on the African continent (6). Global initiatives such as the WHO’s Global Antimicrobial Resistance Surveillance System (GLASS) and the International FAO Antimicrobial Resistance Monitoring (InFARM) system, aim to collect and standardize antimicrobial susceptibility testing (AST) data, promote data sharing and support evidence-based decision-making (9, 10). According to the 2019 WHO GLASS report, only 14 African countries were submitting phenotypic data at that time. While the number of participating countries has increased significantly since then, many still submit data inconsistently, leading to critical gaps in surveillance, evidence generation, and policy formulation.

Whole genome sequencing (WGS) has emerged as a data-rich surveillance technology, providing a high-resolution picture of AMR genetic determinants, evolution, and transmission (11–13). As a surveillance tool, it enables rapid and precise identification and tracking of pathogens for outbreak management. It can contribute to better disease prevention strategies, targeted interventions, improved treatment, and more informed healthcare policies and public health actions provided that data is used and communicated appropriately (14). Additionally, information gathered from multiple time-consuming and expensive phenotypic tests can usually be gained from WGS at a lower cost overall. While phenotypic AST remains essential, WGS provides a complementary and often more comprehensive approach. Recognizing these advantages, institutions like WHO and Africa CDC have begun integrating WGS into their pathogen surveillance systems (15). However, to be effective, genomic surveillance relies on robust bacteriology services, trained personnel with microbiology expertise, and adherence to laboratory best practices to ensure that suitable, reliable and high-quality samples are obtained. The Mapping AMR and AMU Partnerships (MAAP) study by the African Society for Laboratory Medicine (ASLM) recently established that only 1.3% of African laboratories perform bacteriological analysis for priority bacterial pathogens (16) and only 10% meet the required standards for advanced microbiological diagnostics (6). In-country sequencing capacity and investment or support for genomic sequencing of bacterial isolates is also only referenced in a handful of NAPs. As of 2023, only 28% of NAPs had costed and budgeted AMR operational plans, and none had dedicated national budgets (6), largely due to the associated cost and limited appreciation of the value of genomic sequencing (15). Consequently, there is a great opportunity to use WGS technology to leapfrog laboratory limitations and incentivize improvement of microbiology competence (17), contributing to closing the data gap on critical bacterial pathogens and strengthening AMR surveillance systems on the continent.

The SeqAfrica project was established following a call by the Department of Health and Social Care (DHSC)’s Fleming Fund, a UK Aid program, to advance WGS capacity for bacterial AMR across Africa. At the core of the Fleming Fund’s Theory of Change is capacity building, with phase 1 of the program (2017–2023) focusing on increasing data production, improving the quantity and quality of national data. The objectives of the SeqAfrica project are to expand, develop, and consolidate WGS and bioinformatics in Sub-Saharan Africa, with a focus on AMR across all One Health sectors, directly supporting African Union and WHO framework objectives to strengthen AMR surveillance systems, harmonize data generation, and build workforce capacity for One Health implementation. The project also aims to generate data for action *on* the continent *for* the continent. Phase 1 of SeqAfrica launched in August 2019 with the goals of establishing a network of three regional WGS sites in West, East, and Southern Africa to conduct genomic surveillance of AMR and function as a referral system for investigating outbreaks and unusual AMR phenotypes across Africa; sequencing 14,000 bacterial genomes; and providing in-person training of up to 25 individuals from Fleming Fund countries on the use of WGS in AMR surveillance and bioinformatic analysis of sequencing data. In this context, a regional WGS site refers to a sequencing center serving multiple countries by providing WGS services and interfacing with national surveillance systems through specimen referrals, data generation, and feedback mechanisms that support public health decision-making. Here we report on one of the first genomic AMR surveillance networks in Africa, showcasing a model for how to build a network and lift its capacity for WGS and AMR surveillance, bridging and filling the gaps of the AMR landscape on the African continent.

## 2 Establishing a network of regional sequencing centers

**Figure 1:**
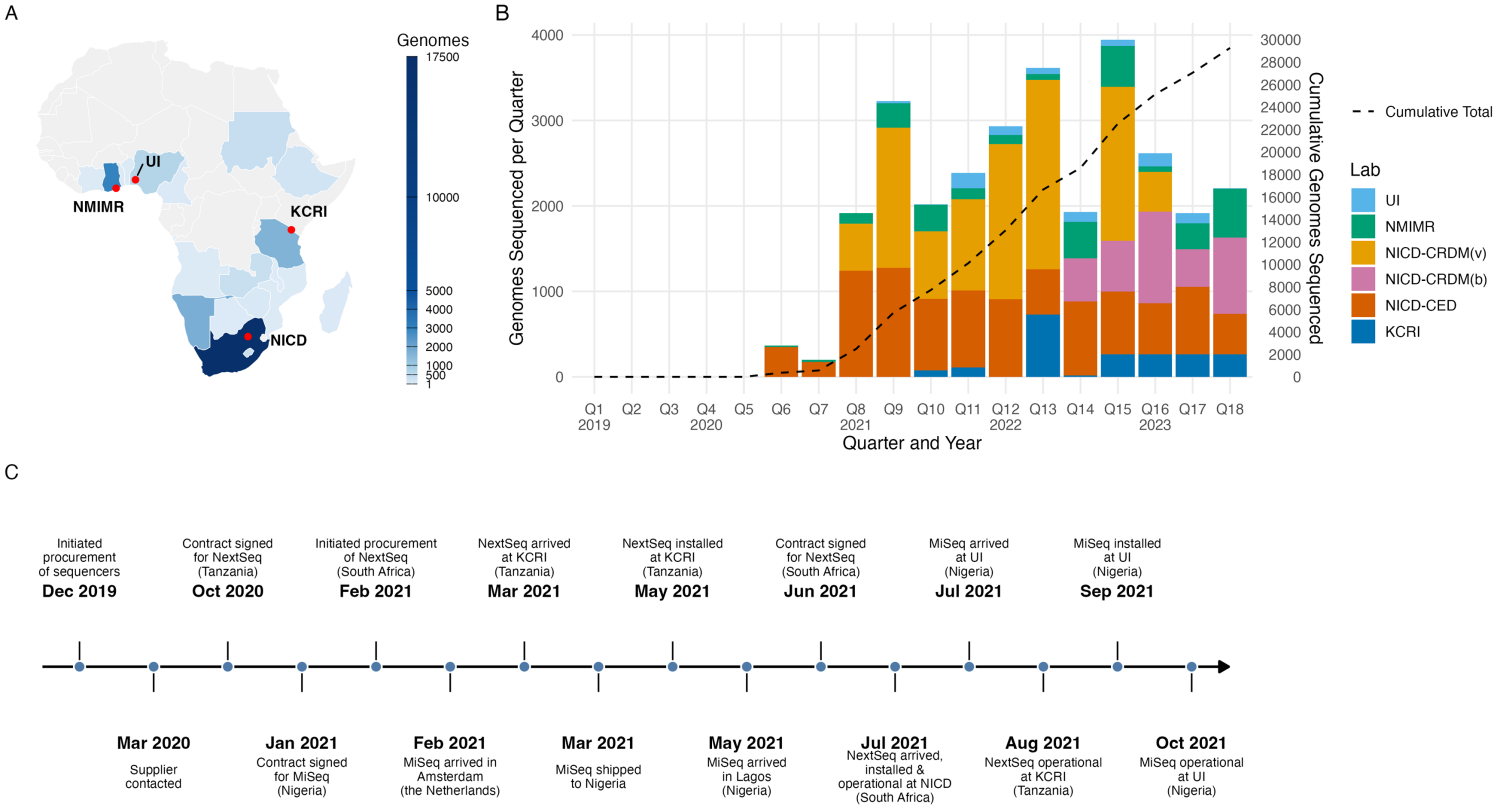
Whole genome sequencing (WGS) in SeqAfrica by regional hubs (2020–2023). **(A)** A chloropleth map of Africa, showing SeqAfrica regional sequencing hubs and the countries of origin for all generated genomes, represented on a color scale. The map includes SARS-CoV-2 genomes. Supplementary Table 1 provides a summary of genomes per country. Regional hubs were located at Noguchi Memorial Institute for Medical Research (NMIMR) in Ghana; University of Ibadan (UI) Nigeria; Kilimanjaro Clinical Research Institute in Tanzania; and the National Institute for Communicable Diseases (NICD) in South Africa, which hosted the Center for Enteric Diseases (CED) and Center for Respiratory Diseases and Meningitis (CRDM). **(B)** WGS by partner by project quarter. The graph includes the cumulative total sequencing throughout phase 1 (2019–2023). NICD-CRDM (v) and NICD-CRDM (b) indicate SARS-COV-2 and bacterial genomes generated at CRDM, respectively. The project’s inception period concluded in quarter 4, coinciding with the onset of the SARS-CoV-2 pandemic, which delayed the initiation of sequencing and affected sequencing efforts, particularly in the early stages of implementation, though the impact diminished in later quarters. **(C)** Timeline of sequencer procurement, installation and operationalization across SeqAfrica regional hubs. Events represent key milestones in the acquisition and deployment of Illumina sequencing platforms (MiSeq and NextSeq) between 2019 and 2021 across Tanzania, Nigeria, and South Africa.

To function as a referral system for AMR sequencing servicing national and international surveillance efforts and individual clients, a network of regional WGS reference centers (called regional hubs throughout the paper) was established. A limited number of countries in Africa had operational next-generation sequencing (NGS) platforms in 2019, even fewer had comprehensive sequencing facilities, and sequencing was primarily deployed for research purposes at research facilities or other academic institutions (18, 19). Regional level service rather than individual local laboratory sequencing across the 14 Fleming Fund priority countries in Africa (20) was chosen as the most cost-effective and sustainable solution to provide sequencing access to countries. This was due to the size of the economic investment in WGS platforms and IT infrastructure, and the economy of scale to be gained through bulk processing and high-throughput sequencing at regional centers. SeqAfrica first sought to identify prospective regional sequencing sites by scoping within established collaborations and contacts in the AMR field, looking for existing capacities upon which a regional network could be built. Sites were selected based on a structured capacity assessment within these collaborations, ensuring a minimum level of capability, including skilled staff in microbiology, sequencing and bioinformatics, access to sequencing equipment, and availability of relevant samples. SeqAfrica sought to include sites with diverse and complementary specialties based at either public health institutions or research facilities and include sites with varying degrees of capacity. The initial network included the Department of Pharmaceutical Microbiology, University of Ibadan (UI) in Nigeria; Kilimanjaro Clinical Research Institute (KCRI) in Tanzania and the National Institute for Communicable Diseases, Center for Enteric Diseases (NICD-CED), tasked with servicing West, East and Southern Africa, respectively. The consortium also consisted of a coordination and management hub, and technical lead at the Technical University of Denmark (DTU), National Food Institute in Denmark. The three regional hubs all had existing skills in WGS of bacterial pathogens and AMR, genomic data analysis, and data storage. All sequencing teams also worked proactively to establish or strengthen links with existing surveillance systems, to ensure access to relevant specimens for sequencing. Medium- to high-throughput sequencing capacity was already established at KCRI and NICD. NICD was the most well-equipped and advanced site, possessing a separate Sequencing Core Facility with a range of short- and long-read WGS platforms and an established national laboratory surveillance system (GERMS-SA) for bacterial and fungal pathogens. At inception, KCRI reported receiving a moderate number of common bacterial pathogens per year and a moderate annual sequencing throughput of a few hundred isolates (21–24), while UI was starting routine sequencing of key bacterial pathogens on a lower-throughput scale. In early 2020, the network launched a second West African site at the Noguchi Memorial Institute for Medical Research (NMIMR) in Ghana. This site first served as a pilot national sequencing center, initiating a genomic surveillance program targeting WHO’s GLASS priority pathogens, graduating to a regional hub as the project evolved. Several hospitals nationwide were enrolled and tasked with submitting isolates to NMIMR for WGS, thereby enhancing the Ghana’s ability to monitor and respond to these critical health threats. In July 2020, in reaction to the global SARS-CoV-2 pandemic, the project redirected some sequencing efforts to support the global genomic surveillance of SARS-CoV-2 via the inclusion of a second laboratory at NICD (Center for Respiratory Diseases and Meningitis (CRDM)) dedicated to WGS of SARS-CoV-2 in South Africa and neighboring countries. This addition leveraged the Africa CDC’s pandemic response support in the Southern African Development Community (SADC) countries with additional sequencing of samples. Furthermore, SeqAfrica supported NMIMR in sequencing SARS-CoV-2 genomes to contribute to national efforts in tackling COVID19 in Ghana during the pandemic. Finally, bacterial causes of pneumonia and meningitis in GLASS were also sequenced through NICD-CRDM. Ultimately, SeqAfrica strengthened and equipped a network of five regional sequencing hubs across Africa (Figure 1A). The network of laboratories at different stages of implementation enabled continued exchange of local experiences in developing new or extended capacities and provided support and solutions to existing or emerging barriers and challenges. It also provided flexibility to redirect sequencing requests from external clients to other partners when a regional hub experienced sequencing delays or prolonged interruptions due to procurement or technical issues.

## 3 Scaling up sequencing infrastructure

SeqAfrica first prioritized expanding genomic sequencing capacities and capabilities at the regional sequencing sites through procurement of additional medium- and high-throughput short-read sequencers, auxiliary sequencing equipment, and training of staff at the regional hubs in sequencing techniques and bioinformatic analysis. The regional hubs at UI in Nigeria and KCRI in Tanzania required new or upgraded sequencing instrumentation to increase their capacity to meet the project’s sequencing goals. Following a needs assessment, the SeqAfrica project management team at DTU initiated procurement processes at the start of 2020 with the, at that time, sole supplier of short-read sequencers for Africa. It took 8 and 11 months to finalize negotiations of terms and conditions related to legal obligations, payment and service agreements (Figure 1C). Another 9 to 10 months passed before the two sequencing machines were delivered, installed, and operational. The management team faced multiple challenges with shipping processes and customs clearances, and eventually the project resorted to purchasing services from a professional procurement agency (International Procurement Agency, IPA). Contributing factors to these extensive lead times, which disrupted procurement throughout phase 1 of the project, included: (1) supply chain restructuring by the sequencing equipment manufacturer at a crucial point in the procurement process, (2) a lack of local representation from the manufacturer on the African continent and the resultant dependency on intermediaries for procurement, (3) no in-house or on-continent stock, and (4) travel restrictions caused by the COVID-19 pandemic, which prevented technical service engineers from installing the equipment. Having no local service engineers from the provider in Nigeria or Tanzania meant that equipment sat idle in the laboratories for extended periods, delaying progress for these partners. Indeed, the onset of the COVID-19 pandemic caused global disruption of established supply chains, severely affecting already fragile systems and procedures. Demand for sequencing reagents and basic molecular biology consumables like filtered pipette tips also increased across the globe as SARS-CoV-2 sequencing increased, thus contributing to delays (25, 26). In contrast to these procurement challenges in Nigeria and Tanzania, subsequent procurement of a high-throughput sequencer for NICD in South Africa in 2021 to increase sequencing throughput to meet the demands of SARS-CoV-2 genomic surveillance only took 6 months from initiation until the machine was operational (Figure 1C). This discrepancy highlights differences in procurement efficiency between countries such as South Africa, which had well established local suppliers and service engineers, and countries such as Tanzania and Nigeria, where such infrastructure was limited at the time. Stock-outs or inaccessibility of staple consumables, long lead times and administrative bottlenecks affected all five of the regional hubs at some point during phase 1 implementation, with some hubs experiencing additional or more severe difficulties than others (Box 1). Close engagement with the suppliers of sequencers and reagents was maintained throughout the project, and, alongside other sequencing efforts on the continent, paved the way for more local representation and restructuring of supply chains to improve procurement processes for WGS in the region. While in severe cases, SeqAfrica arranged procurement at DTU in Denmark and subsequently shipped the needed equipment and materials to the sequencing sites in Africa this is not a sustainable solution. By procuring equipment and reagents on the continent, SeqAfrica hopes to be part of building the needed demand to entice suppliers and manufacturers to locate offices and factories in Africa. Therefore, the prevailing practice of the project was to purchase items through the regional sequencing centers even though this sometimes caused great delays, higher costs, and extra work.

Box 1 **Infrastructure challenges in genomic transition**

The SeqAfrica regional hub at UI in Nigeria experienced some unique procurement and operational challenges transitioning from a conventional bacteriology laboratory performing some molecular work into an operational genomic surveillance hub. Continuous needs assessment revealed the need for various auxiliary equipment ranging from typical molecular biology equipment that would boost sequencing throughput, equipment for cold storage in the laboratories to contain bulk orders of reagents, and solar driven back-up power system to handle the power requirements from sequencing machines due to the unstable national power grid system. The cost of this additional equipment was approximately 50,000 USD, with the solar-powered back-up system alone accounting for nearly 48,000 USD (based on 2021 NGN to USD conversion rates).

## 4 Strengthening genomic skills through hybrid learning

**Figure 2:**
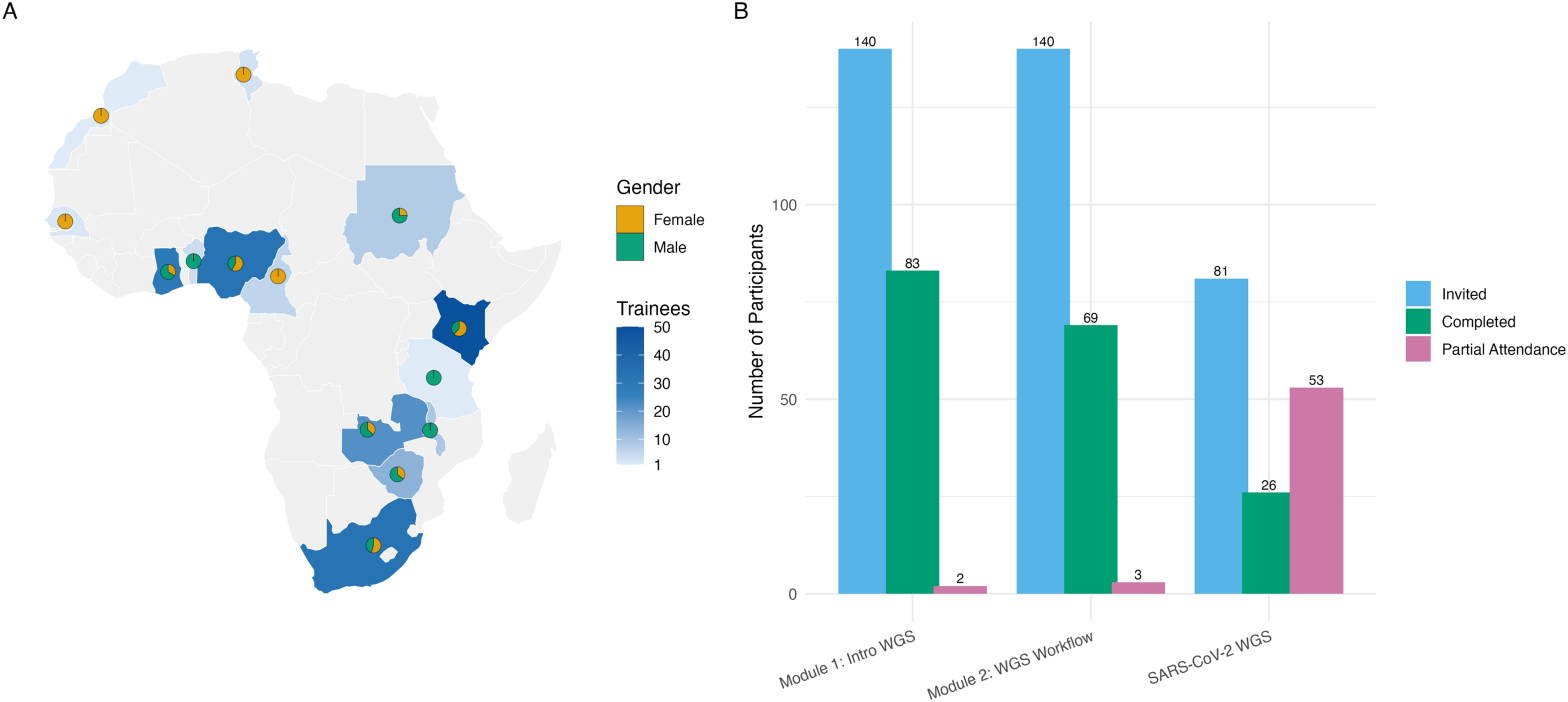
Online training reach and participant engagement across SeqAfrica modules 1–3. **(A)** A cholopleth map of Africa, illustrating training coverage of the three online modules using a color scale. The overlayed pie-charts at the country’s centroid represents the female-to-male gender distribution. The overall gender distribution was 50.7% female. **(B)** A grouped bar plot showing the number of participants invited, completed, and with partial attendance for each module: Module 1: Introduction to whole genome sequencing for AMR surveillance (February 2021), Module 2: WGS workflow: from isolate to analysis (March 2021), and Module 3: SARS-CoV-2 whole genome sequencing (May 2021). The high number of partial attendances in Module 3 reflects participants who attended all sessions but did not submit the analysis summary report required for course completion.

Training scientist and laboratory workers in how to operate equipment and generate, analyze, and correctly interpret sequencing data is as important as procurement (27, 28). The SeqAfrica project initiated a training program for its five regional hubs, and for personnel from laboratories in other African countries. South–south training for young researchers and professionals was prioritized to build training and leadership skills, support sustainability, and ensure that training was best tailored to the prevailing conditions and context of the trainees. The in-person training of regional partners in February 2020 was focused on Oxford Nanopore Technologies (ONT) long-read sequencing techniques and advanced bioinformatics. This training-of-trainers was designed so that partners would be well-equipped to teach a more extensive in-person training course, which would have introduced core principles of NGS technologies to human and animal health professionals across Fleming Fund-supported countries. However, with the onset of the COVID-19 pandemic, the SeqAfrica team pivoted to instead developing a series of three virtual training courses that covered both the theory and practice of WGS techniques and downstream bioinformatic analysis for first-time and novice users. This shift to online learning allowed SeqAfrica to offer training to a larger and broader audience. The training courses included both live and pre-recorded lectures and were recorded for subsequent distribution and maintained as a freely available resource. Each course included facilitators from the SeqAfrica consortium, international organizations like the WHO and Africa CDC, and private biotech companies (Illumina, ONT and Hyrax Biosciences), which were either based in Africa or elsewhere. Interest in the courses was high, with 350 individuals registering their desire to participate in modules one and two. Through a competitive shortlisting process, which included an entry survey, and prioritization of African and Africa-based professionals, 140 individuals were invited to attend. Classes were spread out over a two-week period providing time in-between classes for participants to complete mandatory quizzes and practical exercises to practice the skills and methods introduced. All presentations, recordings and training material were made available to the participants, either upfront or at the end of each course day, to account for the possibility of poor internet connection or stability. The training team also created a Slack channel for course participants to communicate with speakers and instructors throughout the courses. Sixty-nine animal and human health professionals from 12 countries completed the two-module training program, earning 138 certificates of completion, and a further 14 certificates were granted to additional professionals for completing one of two modules by the end of March 2021 (Figures 2A,B). SeqAfrica developed and launched a third virtual training course specifically for SARS-CoV-2 sequencing and analysis in May 2021. Participants completed an analysis summary report intended for public health officials based on a case study, which included the analysis of pandemic strains and detection of variants of concern. This activity provided the participants with experience in communicating data for action. Of the 81 invited participants, 26 individuals from eight countries completed this training by submitting the final assignment of the summary report (Figure 2B). For all three modules combined, the gender distribution was 50.7% female, with most of the trainees working in the human health sector (80.0%; Figure 2A). NICD-CRDM provided virtual and in-person iterations of the course to an additional 13 key personnel in four SADC countries throughout 2021 and early 2022, significantly enhancing the region’s capacity to respond to the pandemic. During the virtual training for bacterial genomics, the SeqAfrica team identified several scientists and health professionals with the drive or position to have local impact in genomics and bioinformatics. The project invited 25 of these individuals from the human and animal health sectors to an in-person training to refresh their sequencing theory and provide them with hands-on practical wet lab experience covering Illumina and ONT sequencing techniques. This 10-day training took place in October 2022 at NMIMR in Ghana using local infrastructure and SeqAfrica personnel. In May 2023, with co-sponsorship from the FAO, the project invited these same professionals to a three-day in-person refresher course covering ONT sequencing of up to 12 of their own bacterial isolates using MinION devices, and command-line bioinformatics. The FAO donated MinION devices and high-end laptops with specifications tailored to handle MinION sequencing and bioinformatic analysis to the participant’s home institutions so that genomic work could continue post-training. However, despite these efforts, WGS did not continue after the training at most of these sites due to supply chain challenges and severe ONT equipment and reagents delivery delays, with only 40% of trainees receiving their ONT starter packs 15 months post-training. This highlighted the ongoing challenges in sustaining genomic surveillance initiatives without consistent financial and logistical support. In addition to the organized training workshops, continuous peer-to-peer training and professional development occurred at each regional sequencing site among colleagues and students connected to the research groups and institutions. This approach built capacity and capabilities beyond traditional courses, supporting the sustainability of skills in genomics and genomic analysis. Students and early career scientists and health professionals involved in these activities benefited greatly, gaining hands-on experience and mentorship that enhanced their expertise and career prospects in the field. As a mentoring institution in the Fleming Fund Fellowship scheme, DTU connected Fellows with SeqAfrica WGS sites, combining resources and efforts across grant schemes. This integration not only increased value for money by merging activities wherever possible but also significantly enhanced the impact of these connections, thereby amplifying the collective expertise and resources available for surveillance and genomic research across the region. Additionally, SeqAfrica’s support of a bioinformatics scientist at the peak of the SARS-CoV-2 pandemic, who not only led the establishment of the genome analysis workflows, but also coordinated the inter-Africa collaborations during this time, had a significant impact on continental ecosystem strengthening. True sustainability and solidified institutional memory remain a challenge due to workforce retention constraints. Loss of skilled staff was a greater challenge for some regional hubs, with several trained individuals leaving for advanced academic opportunities such as PhD or postdoctoral positions. While this reflects the high demand for skilled professionals, it also underscores the need for strategies to mitigate brain drain, including providing career advancement opportunities as described by Onywera et al. (29). Regional collaboration in genomics is crucial for building a robust and sustainable infrastructure, but genomic and bioinformatics skills also need to be embedded in graduate degrees and programs at more African universities and technical colleges (29). By increasing the critical mass of human resources in this field, the project planted seeds and fueled the drive of local champions who have since acted as catalysts in their fields. This ripple effect is essential for creating a community of scientists and health professionals dedicated to advancing surveillance and genomic research, improving public health outcomes, and protecting food supplies.

## 5 Accelerating genomic data production and access for AMR pathogens in Africa

**Figure 3:**
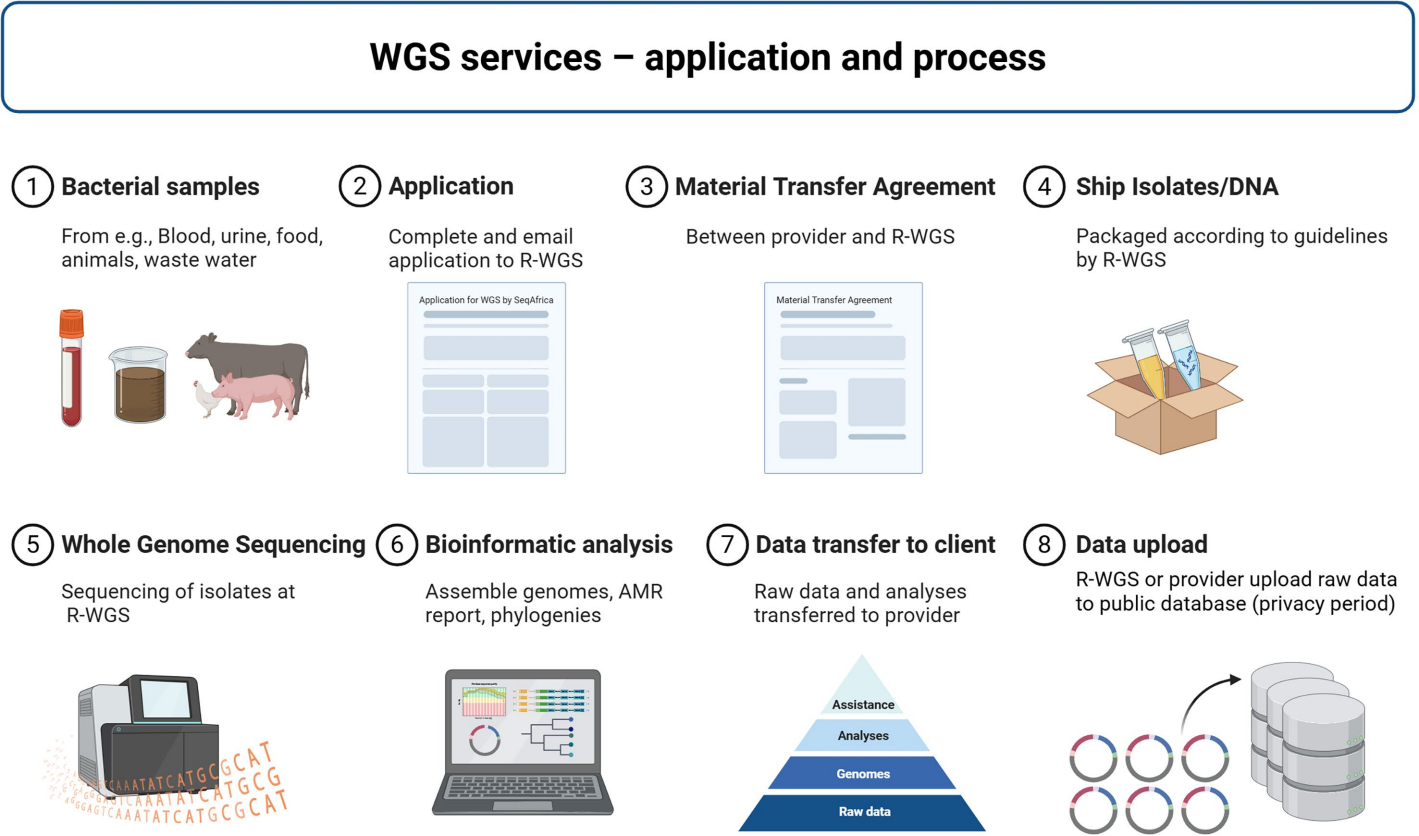
Overview of whole genome sequencing services and process. R-WGS: Regional sequencing hubs. Available samples (1) from human clinical sources, animals, food, or the environment, were considered for WGS through an application (2) submitted to the regional hubs. If accepted, a Material Transfer Agreement (MTA) was negotiated between the data owner (client/provider) and the regional hub (3). Once the MTA was countersigned, clients shipped isolates or extracted DNA to the regional hub (4). The regional hub conducted quality checks and proceeded to WGS (5) and bioinformatic analyses of the sequencing data (6). Raw data, genome assemblies, and analysis results were then transferred to the client, accompanied by a report and the option for an online feedback session (7). Clients were encouraged to upload sequencing data to public repositories, with assistance available from the regional hubs (8). Regional hubs could also upload data on behalf of the clients and offered a privacy period of up to 2 years before public release. Created in BioRender. Nilsson, P. (2025), https://BioRender.com/kl9wmg7

**Figure 4:**
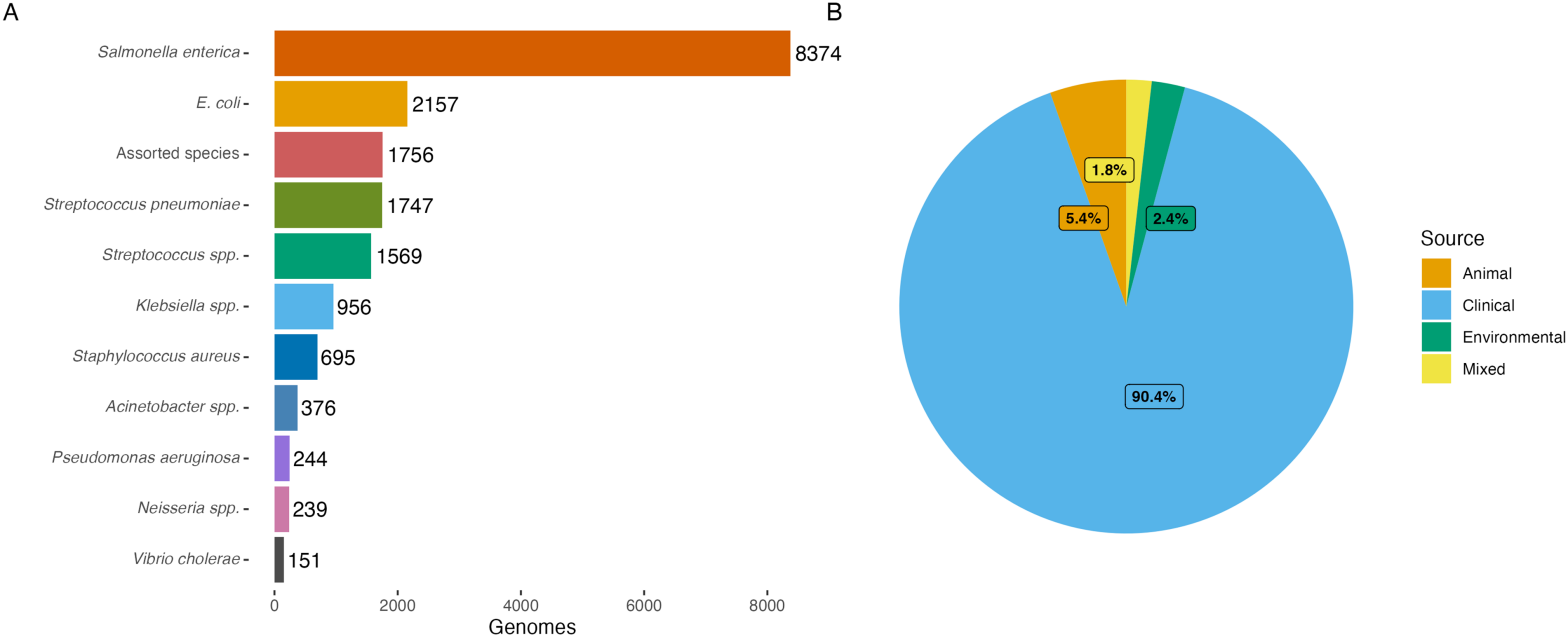
Overview of bacterial genomes generated by regional hubs in phase 1 (2019–2023). **(A)** Barplot showing distribution of bacterial genera of generated genomes (*n* = 18,264). **(B)** One Health source distribution of bacterial genomes (*n* = 18,264), including: human clinical (*n* = 16,510; 90.4%), animal (*n* = 994; 5.4%), environmental (*n* = 432; 2.4%), and mixed (*n* = 328; 1.8%). Mixed sources refer to combinations of two or more sources where further details were not specified in the submitted metadata. All SARS-CoV-2 genomes (*n* = 10,705) were of human clinical origin and were omitted from the plot.

The regional sequencing hubs provided local and national sequencing services of priority pathogens and served as referral sites for clients across Africa, accepting requests from researchers, academic, public, and private institutions as well as international organizations conducting projects on AMR and AMR surveillance. The clients covered the cost of shipping isolates or DNA to the hubs, while SeqAfrica covered all expenses related to isolate processing, WGS and bioinformatic analyses. The scope of these *ad hoc* sequencing efforts was deliberately broad, to introduce the added value of WGS to individuals and groups in the AMR field unfamiliar with the technology and allow scientists with limited funds to take advantage of the technology. Beyond expanding sequencing access, a key goal was to translate data into actionable insights. To this end, SeqAfrica prioritized feedback loops in which clients received not only raw data, but contextualized analysis summaries designed to inform AMR strategies or diagnostic stewardship initiatives. This approach aimed to bridge the gap between data production and real-world utility. The hubs had the flexibility to forward requests to the other hubs in cases where, for example, the pathogen could not be handled at the original hub or if it was an urgent request that could be sequenced more quickly at one of the other centers. The regional hubs used a decision tree to process WGS requests and ensure rational sequencing based on predefined criteria: (i) a reasonable number of isolates per client (typically 50–100), (ii) inclusion of priority pathogens relevant to AMR surveillance (e.g., *Salmonella enterica* Typhi and *Escherichia coli*), (iii) public health utility such as outbreak investigations or atypical AMR profiles, (iv) geographic representation within the region, and (v) emphasis on collections from animal and environmental health sectors to promote One Health balance. Key elements of the WGS service include the negotiation of a Material Transfer Agreement between the client and sequencing hub to formalize the service, ensuring sample and data ownership remains with the provider (Figure 3). This is a fundamental point of the agreement to alleviate mistrust and concerns about exploitation and fairness (30). Upon receipt of the samples, the sequencing center conducted quality checks, including confirmation of bacterial identification, before sequencing the sample. Clients frequently faced challenges with accurate bacterial identification and issues related to sample contamination. This necessitated additional steps by staff at regional hubs to ensure only high-quality samples were processed for sequencing. Sequencing site staff followed up with these clients, providing tailored feedback, education, and guidelines to help them avoid such issues in the future. Upon completion of sequencing, the regional hub provided the client with bioinformatic quality control and basic analysis, raw data, genome assemblies, and any optional analyses requested. Individual online sequencing feedback sessions were offered to all clients to explain the sequencing data and results. The median turnaround time (TAT) from sample receival at hubs to data returned to client was 12 weeks (3–104 weeks). Clients were encouraged to submit raw sequencing data to public repositories or databases and could request assistance from the sequencing center. Data was only submitted in agreement with the provider. The project permitted privacy periods of up to 2 years, allowing time for data processing and manuscript completion before public release. Approximately 30% of clients requested additional assistance with data analysis and interpretation. Regional hubs also provided varying levels of support for scientific publications, ranging from review and editorial feedback to leading or co-authoring manuscript preparation. Consequently, the regional hubs dedicated considerable time and personnel to support these clients, which further strained their resources and prolonged timelines for data dissemination and submission to public repositories. However, this investment in time and resources was not wasted; it played a crucial role in building and solidifying the capacity in the region for WGS and AMR surveillance. By providing comprehensive support, the sequencing centers helped enhance the skills and knowledge of local scientists and health professionals, fostering a more robust and self-sufficient professional community capable of tackling AMR challenges effectively. At the time phase 1 closed in September 2023, SeqAfrica, through its five regional hubs, had collectively generated 29,269 genomes: 18,264 bacterial genomes, 300 fungal genomes, and 10,705 SARS-CoV-2 genomes. The genomes originated from providers in 21 African countries (Supplementary Table 1) and comprise over 120 bacterial species in addition to the SARS-CoV-2 virus (Figures 1A, 4A). All genomes underwent quality control using in-house pipelines implemented at the regional hubs. High-quality bacterial genomes were defined as those meeting or exceeding standardized thresholds (coverage ≥ 20x, completeness > 98%, contamination < 5%). This data has aided pandemic surveillance (31–40), and outbreak investigations of cholera and foodborne diseases (41–43). Extensive genomic data has been generated from major AMR pathogens like *Salmonella enterica* (13, 44–50), ESBL-producing *Escherichia coli* (51–56), *Streptococcus pneumoniae* (57), *S. pyogenes* (GAS), *Staphylococcus aureus* (58–60), *Klebsiella pneumoniae* (61, 62), *Acinetobacter baumannii* (63, 64), *Neisseria meningitidis* (65), *Pseudomonas* spp. (66, 67), and *Corynebacterium diphtheriae* (68). There was limited access to isolates from the animal and environmental health sectors, resulting in 90% of bacterial genomes being of human clinical origin and only 5% and 2% from the animal and environmental health sectors, respectively (Figure 4B). This imbalance stems largely from insufficient resources for collecting and processing samples within these sectors, which could be significantly improved with greater investment. Still, important contributions were made to strengthen One Health surveillance. For example, genomic data were generated for the WHO Tricycle project (69) by sequencing ESBL-producing *E. coli* from all One Health sectors collected in Ghana (70, Egyir et al., in preparation) and through sequencing Fleming Fund Fellow’s samples collected under a Tricycle umbrella in Ghana and Nigeria (51, 70). In addition, SeqAfrica enabled sequencing of environmental isolates from hospital wastewater in Nigeria (63), thereby expanding the sparse One Health data pool in West Africa. At the time of writing this manuscript, 82% (100% of SARS-CoV-2, 70% of bacterial) of the genomes had been uploaded to semi-public or public repositories (see Data Availability), including the Global Initiative on Sharing All Influenza Data (GISAID), European Nucleotide Archive (ENA), and National Center for Biotechnology Information (NCBI) GenBank. A subset of the remaining 18% of generated genomes will be uploaded to public repositories as manuscripts progress, however a few of the genomes will never be published due to the lack of associated minimal metadata, or inadequate sequence quality. With the data generated by SeqAfrica in phase 1, 40 scientific manuscripts have been published to date, and more are in progress. Data generated by SeqAfrica’s regional hubs have been used to inform health care and food safety actions both locally and nationally in countries like Ghana, South Africa, and Tanzania, and informed global pandemic prevention and policies (Box 2).

Box 2 **Data-to-action use cases**

Case 1: Routine WGS at NICD of human *Vibrio cholerae* O1 isolates has explained the epidemiology of cholera in South Africa and directed epidemiological investigations (41, 42). Notably, in 2023 NICD-CED investigated imported cholera cases from Malawi and demonstrated that these isolates belonged to the seventh cholera pandemic El Tor sublineage AFR15. This finding confirmed that the cases were not a resurgence of a strain previously circulating in Eastern or Southern Africa, or elsewhere in Africa, but rather represented a new introduction of the cholera agent into Africa from South Asia (42).

Case 2: Support provided by SeqAfrica to NMIMR enabled the establishment of genomic surveillance of GLASS priority pathogens in Ghana. With project assistance, NMIMR initiated systematic collection and sequencing of bacterial isolates from more than 10 hospitals nationwide. This effort not only expanded Ghana’s genomic capacity but also catalyzed the creation of an Antimicrobial Stewardship Committee at St. Martin’s De Porres Hospital, which now oversees antimicrobial use, monitors resistance trends, and guides prescribing practices.

## 6 From insight to action—evolving SeqAfrica’s strategy

SeqAfrica’s phase 1 unfolded in two key stages: an inception period focused on establishing partnerships, expanded infrastructure, and training, followed by an implementation period dedicated to scaling sequencing efforts and regional engagement to maximize genomic data generation. These experiences provided insights into the challenges and opportunities of genomic AMR surveillance across the continent. The establishment and maintenance of a regional sequencing network emerged as a cornerstone of the strategy. Timely turnaround of sequencing is essential for effective genomic AMR surveillance and outbreak detection, as rapid sequencing and data analysis enable prompt cluster identification and identification of resistance patterns, which is critical for informing treatment decisions, public health interventions and outbreak response. The more established site at NICD was able to maintain continuous sequencing with minimal interruptions and shorter TAT from sample receipt to data release (2–3 weeks compared to delays of up to more than 2 years), demonstrating its robust infrastructure and operational efficiency (Figure 1B). The prolonged TAT at the remaining regional hubs were caused by a confluence of factors, in majority related to supply chain issues exacerbated by the COVID-19 pandemic. One of the key insights was the importance of high-quality metadata, which provides essential context for interpreting genomic data, enabling accurate tracking of resistance patterns and informing effective public health actions. In resource-limited settings, effective metadata practices like standardized data collection protocols, registration or access to comprehensive sample information including minimal metadata such as date, location and source of isolation, might not be appropriately implemented due to the absence or dearth of data collection tools and human resources, among other reasons (71). The regional hubs spent considerable time trying to collect the minimal metadata to enable the most insightful data interpretation and data sharing in public repositories. Furthermore, an appreciable number of isolates received for sequencing were incorrectly identified or impure. Based on these observations the project embedded bacteriology strengthening packages, covering culture, AST, and quality assurance and quality control practices in client communication and feedback loops to ensure WGS was built on robust microbiological foundations. Despite these obstacles, the SeqAfrica intervention consolidated capabilities, expanded genomic surveillance capacity, and strengthened collaboration among regional hubs, contributing to a more resilient and self-sufficient network of sequencing centers across the region. Further strengthening in surveillance strategies and outbreak responsiveness is required to enhance overall effectiveness of genomic AMR surveillance. Equally important were the strategic partnerships formed during phase 1. Collaborations with initiatives such as those initiated by the Africa CDC, FAO, and WHO proved instrumental in aligning efforts, avoiding duplication, and leveraging shared resources to maximize impact. Another key lesson was the need to invest in people. Retaining skilled professionals, especially bioinformaticians, required more than training –it demanded clearer pathways and opportunities for growth to counter the acute brain drain experienced.

With these foundational lessons in mind, SeqAfrica transitioned into a more operational and impact driven phase in October 2023. Phase 2 (2023–2026) focuses on consolidating capacities, refining strategies, and translating lessons into targeted actions. The aim is to strengthen surveillance systems further through deeper collaboration, enhanced technical support, and a sharper focus on generating actionable data (Table 1). During phase 2, the project will sequence over 9,000 bacterial genomes from prioritized pathogens or diseases. This data will directly support national and regional priorities by focusing on critical drug-bug combinations and actionable insights. By introducing long-read sequencing into six frontline labs across three countries, it brings diagnostics closer to the point-of-care and fosters real-time data use for infection prevention control and outbreak response in the human and animal health sectors. To ensure long-term impact, phase 2 also prioritizes workforce development and retention through training, mentorship, and a bioinformatics community of practice. It also strengthens sustainability by embedding genomic surveillance into national systems, expanding local capacity, and aligning closely with national and regional initiatives. Looking ahead, sustainability remains a priority for phase 2, with efforts to explore models such as pooled procurement and integration of genomic surveillance into national systems and budgets to reduce reliance on donor funding (Table 1).

**Table 1:** Insights from phase 1 (2019–2023) and how they have shaped the design and priorities of phase 2 (2023–2026).

Key insight	Context and implementation details
1. Focus sequencing efforts on critical pathogens to build data for action	In phase 1, we focused on increasing the amount of available genomic AMR data from the African continent. In phase 2 we have narrowed the pathogen focus and put increased emphasis on generating actionable data relevant and specific to individual country and client contexts, priorities and for critical drug-bug combinations. We have worked more closely with policy makers, engaging directly with national public health facilities with governmental endorsement. The introduction of a continent-wide prioritization framework for high-impact drug-bug combinations, informed by local epidemiology, WHO and FAO priority lists, and national AMR data, would further help standardize pathogen selection and enhance the strategic impact of sequencing efforts.
2. Enhance data sharing through mandatory minimal metadata requests and ENA Umbrella study creation	Data sharing has been streamlined with the Umbrella Study, allowing skilled bioinformaticians to submit sequencing data on behalf of providers while maintaining proper ownership and acknowledgement of the providers. A standardized minimal metadata template has been developed, and no sequencing has been initiated without the requested associated metadata.
3. Bring WGS to frontline diagnostics	In phase 2, the project has expanded by decentralizing sequencing with the creation of sentinel sites, which use portable devices (ONT MinION). The belief is that building genomic AMR surveillance capacity at all levels of the healthcare system and engaging practitioners facilitates greater buy-in, addressing AMR locally and quickly to inform treatment and potentially leads to data-driven policy actions.
4. Strengthening the foundation for sustainable genomic AMR surveillance	The provision of comprehensive support to clients and countries in data analysis and interpretation has continued in phase 2 to further strengthen the foundation for future robust genomic AMR surveillance programs in Africa.
5. Closer collaboration with Fleming Fund Country and Fellowship grant schemes	In phase 1 we provided introductory training, mentorship and WGS services. In phase 2, the collaboration has been further formalized through targeted technical assistance to support country plans and needs including in-person training, greater WGS efforts, and bioinformatics support and mentorship.
6. Leverage major networks for coordination and continuity	In phase 1 we sought to establish collaboration and coordination with emerging initiatives and to maintain established relationships to avoid duplication of efforts and combine strengths and resources when possible. In phase 2 these efforts have continued, and collaboration is solidified by integrating regional sequencing centers into existing networks, such as the Africa CDC Pathogen Genomics Initiative, for enhanced continuity.
7. Build demand for Africa-based suppliers of materials and equipment and working with vendors to push for policy changes	The rapid increase in instrumentation and capabilities in molecular and genomic works in the wake of the COVID-19 pandemic has contributed to the creation of the critical mass needed for changes in supply chains and logistics on the continent. Some laboratories are better equipped to purchase in bulk, and more suppliers now have local presence, although they are still heavily dependent on production and storage facilities based outside Africa. Procurement lessons from phase 1 have emphasized the importance of being creative and resourceful. In phase 2, we have continued to build procurement capacity within our network, prioritizing local procurement and working with vendors to push for policy changes.
8. Continuing training using distance learning technologies	The demand for specialists in Africa is great and as brain-drain challenges endure continued in-depth training is still in high demand. In phase 2, training topics include genomic epidemiology and outbreak detection, and webinars on selecting isolates, identifying important metadata to maximize genomic insight, and translating data into actionable insights for decision-makers.
9. Provide professional development opportunities for young scientists	Skilled staff, especially bioinformaticians, are hard to keep as their competencies are in high demand. Opportunities for professional development and career growth often present themselves outside their institution, country or continent. To alleviate issues with staff turnover, phase 2 has introduced initiatives to entice young scientists to invest in careers within their countries. Specifically, bioinformaticians at the regional sequencing hubs have conducted exchange visits to enhance and solidify their skills and expand their professional network.

In conclusion, the SeqAfrica project has established a network and framework that significantly enhances the capacity for genomic AMR surveillance across Africa. By fostering collaboration among regional sequencing hubs and integrating advanced technologies, SeqAfrica has built a flexible community dedicated to combating AMR. This initiative has not only provided critical data for action but has also inspired and empowered a new generation of young researchers and professionals. These individuals are now equipped with the skills and knowledge to drive the future of genomic AMR surveillance and public health interventions. Through comprehensive training programs and continuous peer-to-peer support, SeqAfrica has sown the seeds for sustainable and applied genomic AMR surveillance, ensuring that the fight against AMR will continue to evolve and expand. Yet, maintaining this capacity for genomic surveillance remains uncertain in the face of global health funding constraints and are vulnerable to erosion without long-term support. Nonetheless, the project’s emphasis on building local capacity and fostering aspirations among young scientists has laid a strong foundation for ongoing innovation and implementation, ultimately contributing to improved health outcomes across the African continent. As Africa continues to invest in genomic health infrastructure, SeqAfrica provides a proven, scalable model for embedding pathogen genomics into national and regional surveillance frameworks, including collaborations with public health institutions and research centers.

## 7 Limitations

This study has several limitations. First, the project lacked baseline indicators to measure impact beyond activity outputs. While deliverables and milestones served as operational key performance indicators, they did not allow for statistical benchmarking of surveillance outcomes or assessment of long-term effectiveness. Second, the genomic data generated were predominantly from human health sources, with limited representation from animal and environmental sectors, which constrains the One Health scope of AMR surveillance. Third, TAT varied widely across regional hubs. While NICD maintained a 2-3-week TAT enabling near real-time surveillance, other hubs experienced delays of several months to over 2 years, limiting timely data use for outbreak response. Finally, sustainability challenges persist, including reagent and consumable procurement bottlenecks, workforce retention, and dependance on donor funding with limited integration of genomic surveillance into national budgets.

## Data Availability

Sequencing data published to date by regional hubs and WGS clients can be accessed through the following accession numbers: PRJEB39546, PRJEB39739, PRJEB39740, PRJEB39988, PRJEB58695, PRJEB66434, PRJEB68050, PRJEB71118, PRJEB71714, PRJEB71932, PRJEB78366, PRJEB78367, PRJEB78368, PRJEB78369, PRJEB78370, PRJEB78444, PRJNA642017, PRJNA820630, PRJNA835871, PRJNA851427, PRJNA851944, PRJNA860607, PRJNA906096, PRJNA929018, PRJNA950924, PRJNA952500, PRJNA994298, PRJNA1045648, PRJNA1293373, PRJNA1082310, PRJNA1154600 and PRJNA1205738. All SARS-CoV-2 genomes were submitted to GISAID in real-time during the pandemic. All genomes of enteric pathogens from the GERMS-SA Laboratory Surveillance Network in South Africa were submitted to Enterobase in near real-time.
