# Supplementary material for: SeqAfrica: empowering Africa’s fight against antimicrobial resistance through genomics
Source: Front Public Health. 2025 Dec 17;13:1716498. doi: 10.3389/fpubh.2025.1716498 (PMC12753507; doi:10.3389/fpubh.2025.1716498)
Supplement: Supplementary file 1 [file Table_1.DOCX]

**Supplementary Table 1:** The table summarizes the origin of the generated genomes per country and was used as input for the chloropleth map of Africa in Figure 1A. The table summarizes both bacterial and SARS-CoV-2 genomes.

| **Country** | **Genomes** |
| --- | --- |
| Angola | 27 |
| Benin | 32 |
| Botswana | 43 |
| Cameroon | 51 |
| Côte d'Ivoire | 36 |
| Eswatini | 1,170 |
| Ethiopia | 201 |
| Ghana | 2,957 |
| Lesotho | 297 |
| Madagascar | 49 |
| Malawi | 5 |
| Mauritius | 1,974 |
| Mozambique | 25 |
| Namibia | 1,749 |
| Nigeria | 614 |
| South Africa | 17,490 |
| Sudan | 384 |
| Tanzania | 1,619 |
| Togo | 31 |
| Zambia | 362 |
| Zimbabwe | 102 |
| West Africa | 48 |
| Not on record | 3 |
| **Total** | **29,269** |
